# Inconsistencies in rater-based assessments mainly affect borderline candidates: but using simple heuristics might improve pass-fail decisions

**DOI:** 10.1007/s10459-024-10328-0

**Published:** 2024-04-23

**Authors:** Stefan K. Schauber, Anne O. Olsen, Erik L. Werner, Morten Magelssen

**Affiliations:** 1 Centre for Health Sciences Education, Faculty of Medicine, University of Oslo, Oslo, Norway; 2 Centre for Educational Measurement (CEMO), Faculty of Educational Sciences, University of Oslo, Oslo, Norway; 3 Department of Community Medicine and Global Health, Institute of Health and Society, University of Oslo, Oslo, Norway; 4 Department of General Practice, Institute of Health and Society, University of Oslo, Oslo, Norway; 5 Centre for Medical Ethics, Institute of Health and Society, University of Oslo, Oslo, Norway

**Keywords:** Rater-based assessment, Expert judgment, Rater inconsistency, Heuristics, Borderline candidates

## Abstract

**Introduction:**

Research in various areas indicates that expert judgment can be highly inconsistent. However, expert judgment is indispensable in many contexts. In medical education, experts often function as examiners in rater-based assessments. Here, disagreement between examiners can have far-reaching consequences. The literature suggests that inconsistencies in ratings depend on the level of performance a to-be-evaluated candidate shows. This possibility has not been addressed deliberately and with appropriate statistical methods. By adopting the theoretical lens of ecological rationality, we evaluate if easily implementable strategies can enhance decision making in real-world assessment contexts.

**Methods:**

We address two objectives. First, we investigate the dependence of rater-consistency on performance levels. We recorded videos of mock-exams and had examiners (N=10) evaluate four students’ performances and compare inconsistencies in performance ratings between examiner-pairs using a bootstrapping procedure. Our second objective is to provide an approach that aids decision making by implementing simple heuristics.

**Results:**

We found that discrepancies were largely a function of the level of performance the candidates showed. Lower performances were rated more inconsistently than excellent performances. Furthermore, our analyses indicated that the use of simple heuristics might improve decisions in examiner pairs.

**Discussion:**

Inconsistencies in performance judgments continue to be a matter of concern, and we provide empirical evidence for them to be related to candidate performance. We discuss implications for research and the advantages of adopting the perspective of ecological rationality. We point to directions both for further research and for development of assessment practices.

## Introduction

Expert judgment is indispensable, yet infamously inconsistent. Decades of research in areas as different as agriculture, forensics, economics, and medicine support this conclusion (Dror, 2023; Litvinova et al., 2022). In health professions education, experts often function as examiners in rater-based assessment. Here, inconsistencies might impair the trustworthiness of decisions made in such contexts. Generally, inconsistencies in rater judgment in assessment are well-documented (Davis & Karunathilake, 2005; Fernandez et al., 2007; Homer, 2022; Mehic, 2022; Roberts et al., 2000; Sam et al., 2021; Yeates et al., 2013). Even in the case where inconsistencies are small, they still can add up and have larger impacts on individuals, eventually (Teherani et al., 2018). Accordingly, gaining a better understanding of why raters come to different conclusions on identical performance has been a main focus of research in assessment (Gingerich et al., 2011, 2017; Yeates et al., 2019). Simple solutions for handling disagreement, such as to average across performances, have been questioned (Schuwirth & van der Vleuten, 2006). However, there might be other ways to pragmatically resolve the ambiguity that arises from inconsistencies between two or more examiners in a specific situation. This is the issue the current article deals with.

Evidently, discrepancies between raters judgments exist. At least partly, those differences stem from meaningful variations in examiners’ perspectives (Gingerich et al., 2011, 2017). At the same time, others documented the continuing impact of biases (Mehic, 2022; Sam et al., 2021) and differences in rater stringency (Homer, 2021). For example, a strong performing candidate might make a less proficient candidate look even weaker, and vice vera (Yeates et al., 2012, 2013). Importantly, disagreement between rater judgments might be highest where defensibility is most important: in the borderline region between clear passes and clear fails. Observations from standard setting or assessment in postgraduate training echo the phenomenon that such borderline candidates are ambiguous and hence difficult to evaluate (Boursicot, 2006; Boursicot et al., 2021; Gingerich et al., 2020). In conclusion, while inconsistencies might be a consequence of different perspectives, varying standards, or contextual effects, the level of performance the candidate themselves shows can be a major factor contributing to ambiguity of judgment.

One of the few studies that investigates the dependency of agreement in ratings on performance levels comes from cognitive psychology. Here, experts were accurate in recognising excellent performances but were unable to discriminate adequately in the lower end of the performance continuum (Larson & Billeter, 2017). Similarly, a study in medical education suggests highest discrepancies for ratings of borderline candidates; that is, those showing a mid-to-low performance, the minimally competent candidates (Tweed & Ingham, 2010). Descriptively, Holmboe and colleagues document a similar pattern in ratings (Holmboe et al., 2003), while Eva suggests that such large variations are common (Eva, 2018). Surprisingly, there is no study that investigates and tests this relationship using suitable methodology. Still, the literature suggests that evaluating borderline candidates is a difficult, ambiguous task.

How then can we resolve ambiguity from arising inconsistencies, especially in situations that are difficult to evaluate? Current thinking highlights that triangulation of information from multiple sources is essential for making defensible decision on individuals (Schuwirth & van der Vleuten, 2020). Despite these advances, implementing the according extensive systems of assessments in medical schools globally and in due course seems unrealistic. Hindrances might stem from a lack of resources, the need to initiate change in assessment cultures, or a difficulty in fostering assessor readiness (Schuwirth & van der Vleuten, 2020; Tavares et al., 2023). Traditionally, approaches to improve decision making in assessment have been informed by psychometric theory (Schauber et al., 2018). Both rater training and statistical adjustments of scores are possible ways to decrease, or account for, rater variability (Santen et al., 2021). However, also these approaches do not provide a general remedy (Tavares et al., 2023). While possible, adjusting scores statistically does not seem to be generally accepted (Homer & Russell, 2021). Additionally, both approaches require specifically trained personnel, which might, again, not be easily available. However, even if such resources are available, the challenge to adequately resolve potential ambiguity remains. Especially in the face of consequential decisions, it is unclear how a lack of agreement between two or more examiners can be resolved efficiently.

From a global perspective, we assume that many high-stakes rater-based decisions in health professions education are still based on isolated observations of student performance: On conventional or structured viva voce exams (i.e., orals), evaluations of clinical encounters, or OSCEs with a low number of raters and/or stations. With exceptions (Anbarasi et al., 2022; Shrikant & Deepali, 2017; Wu et al., 2022), such practices are rarely highlighted in the scientific literature but are evident, for example, in legal regulations (Approbationsordnung Für Ärzte, 2002 [medical licensing regulation in Germany]). Presumably, the discrepancies in judgments found in the literature reviewed earlier would be tangible in these contexts, too. If our assumption that such assessment contexts are still common on a global level is appropriate, this presses the question of how we can improve decision-making in such environments, too. Put differently, how can we make more accurate decisions with less demands than the approaches delineated above pose.

Current thinking on why between-expert inconsistencies emerge (Dror, 2023) as well as findings from research on human decision making (Dror, 2020; Gigerenzer et al., 2022) might help to address this issue. Clearly, assessment in health professions education often involves decision making. Hence, the according literature could offer a new perspective on a persistent problem. In particular, one line of research, the framework of ‘ecological rationality’, documented that even simple decision rules—heuristics—can boost decision making greatly, especially in realistic, uncertain contexts (Gigerenzer & Goldstein, 1996; Katsikopoulos et al., 2020; Litvinova et al., 2022). This research tradition highlights that fast and simple rules can outperform complex approaches to decision making—given that they fit the context. If they do, seemingly simplistic rules are *ecologically rational*. The goal then is to make decision processes more accurate and/or more efficient. Certain decision rules seem particularly suited for application in rater-based assessment. For instance, some of these heuristics highlight the value of confidence-ratings in decisions. Other decision rules prioritise the evaluations of more experienced decision-makers. Especially promising are approaches that combine independent evaluations by dyads or groups of decision makers, that is, harnessing the ‘wisdom-of-crowds’ (Fifić & Gigerenzer, 2014; Hautz et al., 2020; Kämmer et al., 2017; Kurvers et al., 2016).

## Purpose of the current study

Given the research cited above, we argue that inconsistencies in rater judgment are still a concerning issue. This is especially the case in contexts where resources to mitigate effects of rater discrepancies are limited or where high-stakes decision-making is based on a low number of raters. This matter gains importance if we acknowledge that ambiguity in judging performances is largest where decisions are most consequential, for both, the candidate and society: in the borderline region.

To address this issue, we conducted an empirical, internet-based study where examiners judged video-recordings of varying levels of candidate performance in a mock-exam. Furthermore, we aim to add to the literature on discrepancies in rater judgments both theoretically and empirically. On a theoretical level, we propose that assessment in health professions education might benefit from evaluating the applicability of research in heuristic decision making and wisdom-of-crowds. This is particularly the case for contexts where the possibility to implement robust systems of assessments is currently limited. Empirically, we address two research questions:

- First, we expected that inconsistencies in examiner judgment were related to the level of candidate performance. Our objective was to document the relation between levels of candidate-performance on the one hand and variation in rater-agreement on the other hand in a statistically robust way.
- Second, we want to explore if, and to which extend, applying simple heuristics to the resulting data holds the potential to resolve the insistencies in verdicts.

Taken together, we address a long-standing issue by employing an established theoretical framework to decision making that has, however, never been applied in research on assessment in health professions education before.

## Methods

### Educational context

The current study is set in the context of on an end-of-term exam in the 5th year of a 6 year medical curriculum. The subjects in both the exam and the according module cover general practice, community medicine—including social security medicine and occupational medicine— as well as medical ethics, and health administration and quality assurance.

The assessment context we focus on here is a structured, oral examination using patient vignettes. One exam lasts for about 30 min and examiner pairs conduct the exam and evaluate the performance. The content is developed, and quality assured by the exam board responsible for the module. Typically, eight case-vignettes are developed, and each vignette is used for about 12 students. Before the exam, examiners are provided with slides presenting the clinical cases and a structured description of the questions to be addressed in the exam. Expected correct answers are given as well. In the given assessment context, only pass and fail grades are awarded. The exam is high stakes in the sense that students need to pass the exam to be permitted to move to the next term. There is no specific regional or national licensing exam in the given context: this exam is part of the general licensure process.

### Participants

We defined the study population as appropriately qualified educators who were also otherwise eligible to act as an examiner in the specific oral exam in the given educational context. Inclusion criteria were that, first, the participant was a medical doctor (*cand. med*. in Norwegian). Second, they had to be either experienced examiners in the given exam or they had to be teachers in the relevant module (i.e., without prior assessor experience in this exam). We recruited participants via an email from the regular pool of eligible educators and examiners. Recruitment stopped after we could secure ten participants acting as examiners, in total. Of these, five were experienced, and five others were teachers in the relevant module who had no experience as examiners in the given exam. This sampling distribution was not explicitly intended.

### Materials

The videos of mock exams used in this study were recorded in May 2021. We recruited N = 10 medical students via public announcements distributed through student-representatives’ social media accounts. Students were free to participate in the video study and received vouchers as compensation for their participation. All mock exams were held online and led by two examiners of the exam board and study team (ELW; MM). The Zoom software was used to record the videos.

The oral online exam was structured with a patient vignette and eleven questions which students had to answer and elaborate on. The questions mainly focused on key aspects of a clinical encounter, including history-taking, a clinical investigation, and outlining a treatment plan based on the tentative diagnosis. Each exam lasted approximately 30 min. The video files were stored electronically and handled in compliance with the Norwegian Personal Data Act. Finally, the study team (AOO, ELW, MM) screened the ten recordings and discussed the performances. Two high-performance and two mid-to-low performance videos were selected for further use in the study, with one of the latter including a candidate at the borderline level.

### Procedure

We invited the N = 10 participants to video meetings where a member of the study team (SKS) broadcasted videos of the four pre-recorded mock-exams to the participating examiners. Before the video meeting, participants were provided with the same materials as in the regular administration of the exam, including a document with exam instruction and expected correct (‘best’) answers.

**Fig. 1:**
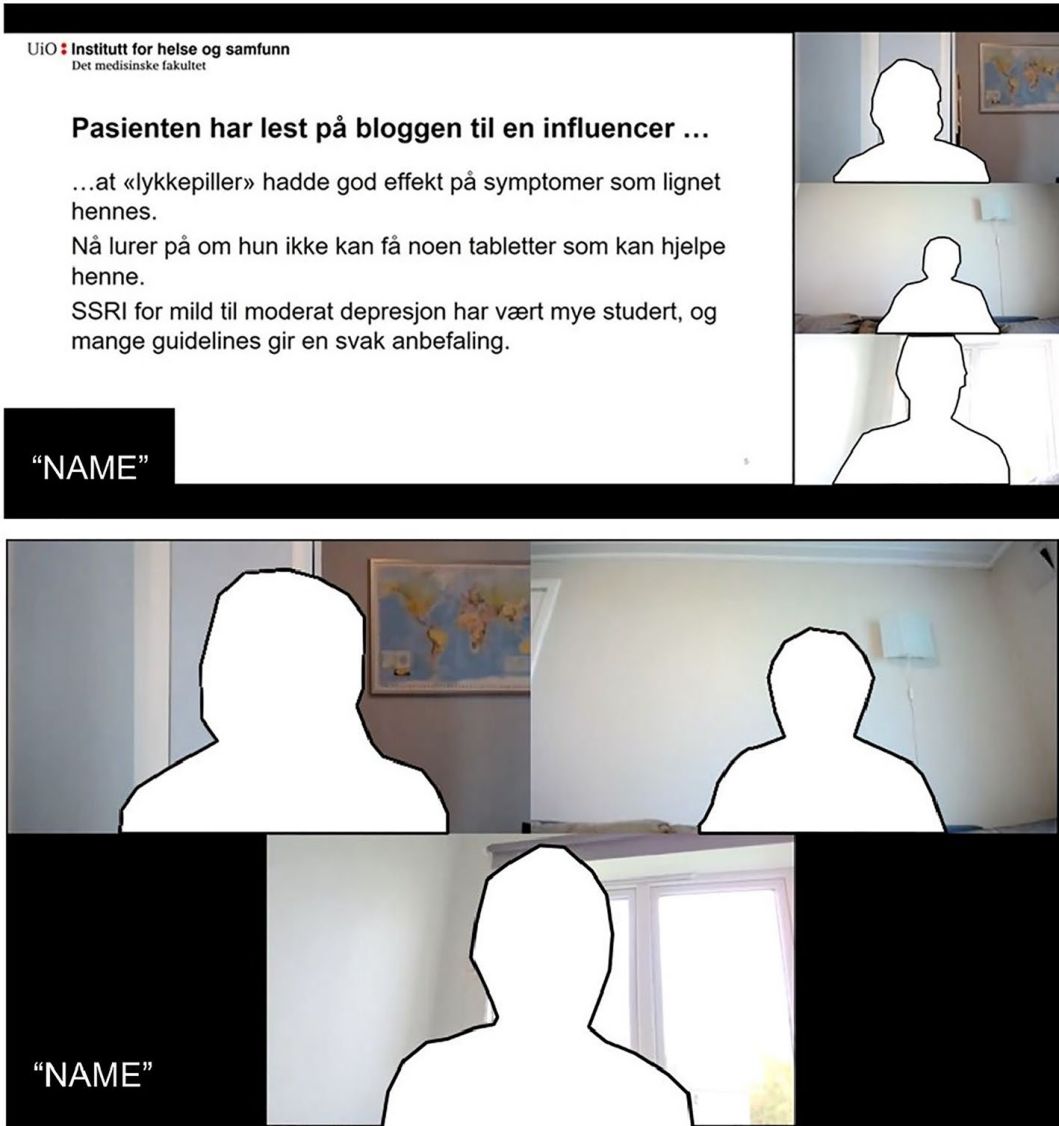
Examples of the Zoom mock exam and the video-ID displayed: Two examiners and one student participate in a structured, case-based oral examination

**Fig. 2:**
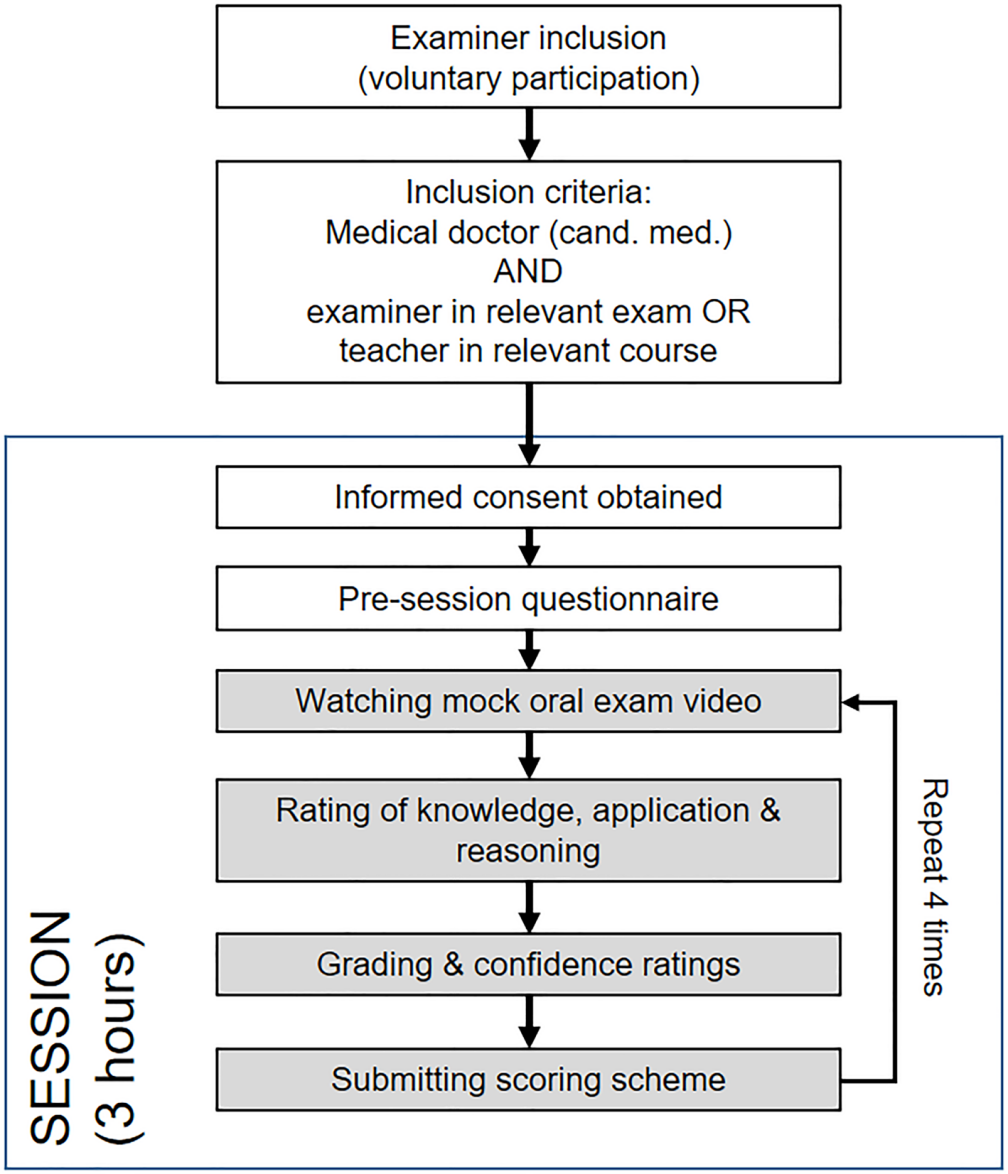
Study procedure—Voluntary participants were included. They were required to be a medical doctor and either an examiner in the relevant exam or a teacher in the relevant courses. A session was scheduled for three hours, and participants saw and rated the performance of four students within this session

Duration of the study for each examiner was about three hours. Each participant rated all four videos independently. However, sessions were either held individually or in a group of up to three participants. In the latter case, participants were instructed not to communicate with each other, either verbally or per direct message. The Zoom software was used to host the meetings and VLC player was used for playing the videos on the host’s computer (cf. Figure 1). Each session started with welcoming the participants followed by a brief introduction to the purpose of the study. We then conducted a quick check of the technical setup, as well as the video and audio quality of the broadcasted videos. The participants were not able to pause or control the video. The host was available throughout the session for questions. The general procedure is given in Fig. 2. Each session started with the same order of videos, with the lower-end performances preceding the higher ones (‘Kari’ → ‘Lars’ → ‘Anne’ → ‘Marit’). Examiners were not made aware of this ordering beforehand. After each video, a link to the scoring rubric was sent via the video-conferencing platform and the participants evaluated the performances according to this rubric.

### Instruments

#### Rating of performance

Examiners classified the candidate as a clear pass, clear fail, or borderline candidate. In alignment with the educational context, we also used the official grading scheme from the University of Oslo, which ranks students on an A to F scale. In this grading scheme, “F” corresponds a fail, while an “E” marks a borderline pass and a “D” a pass with significant deficiencies. An A grade signifies an excellent performance.

Furthermore, we developed a short scoring rubric for this study following the guidance given in the relevant literature (Holmboe et al., 2018). Here, examiners had to evaluate the candidate’s performance on three components. These components were basic knowledge (‘grunnleggende kunnskap’), application of knowledge (‘anvendt forståelse’), and clinical reasoning (‘klinisk resonnement’). Students’ performance in these domains were rated on a five-point Likert-type scale from zero (0, “severe misses/errors”) to four (4, “excellent”). This scoring rubric is given in Table 1.

**Table 1:** Example of the first part of the scoring rubric used in the study

	Severe errors	Multiple errors	sufficient	good	excellent	Not observed
Basic knowledge	□	□	□	□	□	□
Applied knowledge	□	□	□	□	□	□
Clinical reasoning	□	□	□	□	□	□

#### Ratings of confidence in overall grade

Per candidate, examiners indicated their confidence in their own overall A–F grade given on a percent-scale from 0 to 100% in 10-percent steps.

#### Experienced versus non-experienced examines

We defined participants as experienced if they had participated as an examiner in this particular exam before. Those participants who only had taught—but not examined—in the given module were considered non-experienced.

### Data handling

Ratings were recorded using the online survey tool *Nettskjema* (University of Oslo; Norwegian for *webform*) which provides the ability to collect data in a secure manner and according to the European Union’s General Data Protection Regulation (GDPR). Videos of mock exams were stored and handled in compliance with University of Oslo’s guidelines for data with restricted access (‘yellow data’). Processing and analysis of the recorded data was conducted using the R Language for Statistical Computing (R Core Team, 2021).

### Statistical analysis

To address our first objective—dependency of the magnitude of differences on the level of candidate proficiency—we tested differences in agreement for the overall ratings (grades and pass/borderline/fail) and across the four videos. To this aim, we adopted a two-step procedure. In the first step, we defined all possible pairwise-combinations for the participating examiners (i.e., 10 participants = 45 combinations per video/candidate). For all these combinations, we calculated the absolute pairwise differences. If, for instance, for the student named ‘Kari’, examiner one gave a grade of C and examiner two gave an F-grade, then we calculated this difference as F = 6 minus C = 3, hence DIFF = 6 – 3 = 3. We then calculated the average of the 45 pairwise-differences for each video resulting in a total of 180 differences for this sample.

In the second step, we calculated confidence limits for the pairwise-differences using a non-parametric bootstrap procedure. That is, we drew repeated samples with replacement (N = 10,000) from the group of examiners and repeated the procedure described in the first step. The 95% confidence limits were then determined on the basis of the resulting distribution of pairwise-differences. In this way, we tested for statistical significance and were able to investigate our first objective—that examiner agreement was dependent on performance levels.

### Analysis of within- and between-rater consistency

Overall examiner agreement was, for illustrative purposes, also calculated using Fleiss-kappa. According to Landis & Koch, values of Fleiss-kappa at and above 0.60 show substantial agreement, while values of 0.20 and lower indicate slight to poor agreement (Landis & Koch, 1977). Furthermore, we calculated intra-rater consistency as the correlation between the items in the scoring rubric within each rater.

### Establishing an overall verdict for the four candidate performances

Given the lack of a “true” standard or performance level, we considered the combined judgments by all ten independent examiners as a kind of “gold standard”. To determine this group-verdict, we applied the *majority rule* (Kämmer et al., 2017) on the full group of ten examiners. As a robustness-check, we also applied the *majority rule* on the five experienced examiners and the five most confident examiners.

#### Decision rules and heuristics

After we established the verdicts for the four candidates, we investigated to which extent decisions of examiner pairs matched the decisions of this full-group verdict. To this aim, we repeatedly sampled pairs of examiners from the pool of ten and determined if the sampled pair would reach a pass or fail decision by using a heuristic (i.e., decision rule or decision strategy). The heuristics used to arrive at a pass-fail decision for the pairs were based on the *follow-the-most-experienced* heuristic and the *follow-the-most-confident* heuristic (Kämmer et al., 2017) where we chose the more confident or more experience within each randomly selected pair. All heuristics were implemented as an algorithm in the R Language for Statistical Programming.

In total, we compared five decision rules.

**Fig. 3:**
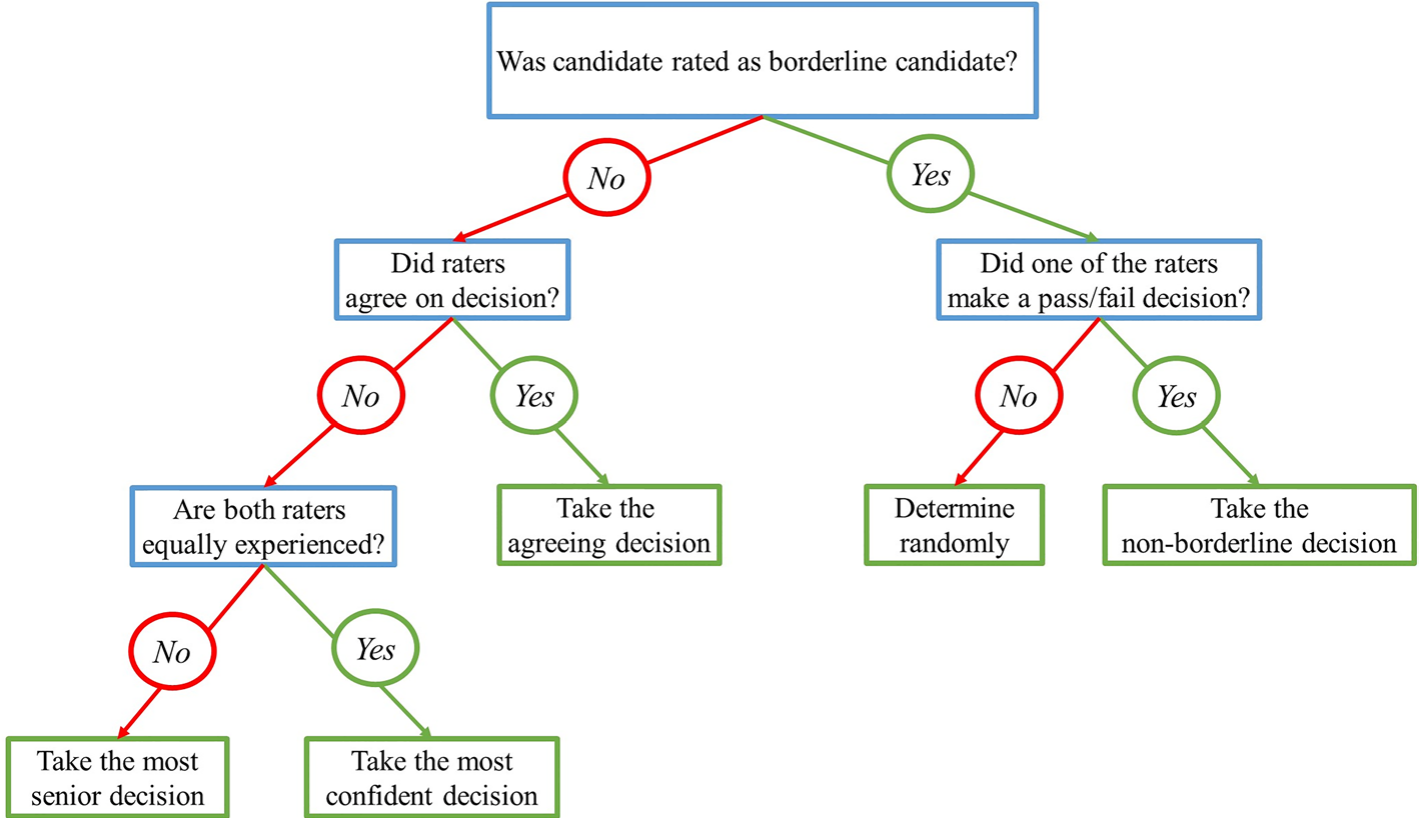
Heuristic decision tree used to determine pass/fail decision. The decision tree was designed from the perspective of resolving borderline judgments into a final pass/fail grading

1. Follow-the-most-experienced.
2. Follow-the-most-confident.
3. Follow the most experienced examiner first. If there were two similarly experienced examiners, choose the more confident.
4. Follow the most confident judgment first. If the verdicts are equally confident, choose the more experienced.
5. We developed a decision tree that integrated the previous strategies. The starting point for the decision rule was to resolve a borderline-decision into a pass or fail verdict. Details on this decision tree are given in Fig. 3 while Luan et al. (2011) give a more in-depth account of this approach.

For all five decision strategies, if the outcome was still “borderline candidate”, the final decision (pass/fail) was randomly chosen. For each rule we repeated the procedure with a total of N = 10.000 pairs since parts of the rules employed a merely random decision. We opted for this approach since the algorithms all included an element of resolving ambiguity by chance (i.e., randomly selecting a decision from the pair).

We compared variation in the different heuristics but also established a benchmark-condition where we selected the final verdict from the randomly chosen examiner pair randomly, without any heuristic applied. This mimics a situation where there is no insight or guidance in the decision process.

#### Ethics and dissemination

The study was evaluated by the Norwegian Centre for Research Data (NSD; Reference number: 812259). Informed consent was obtained from both the students participating in the mock-exams and the teachers and examiners participating in the main study. Participation was voluntary.

## Results

### Descriptive statistics

**Fig. 4:**
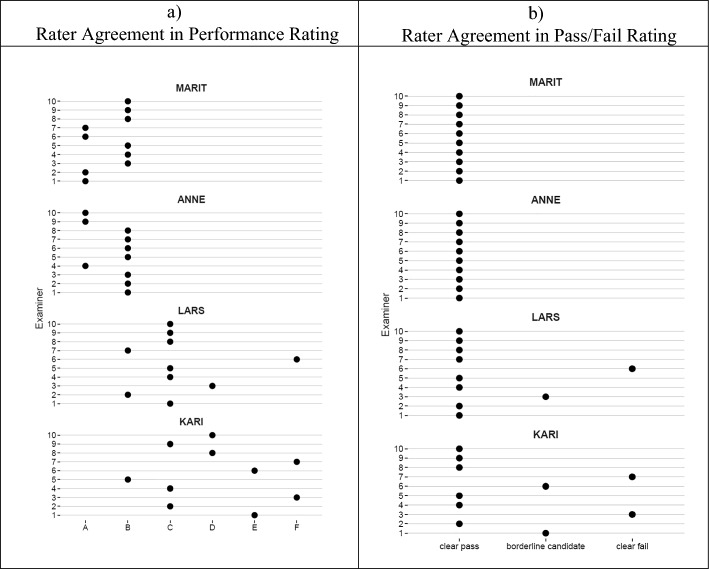
Rater agreement across the ten examiners and four candidate videos. The y-axis shows examiner number from one to ten, the x-axis shows the grades on an A–F scale (**a**) and the pass-borderline-fail classifications (**b**)

Of the total of N = 10 participants in the study, 50% were female. To reduce the possibility personally identify individual participants, we did not collect further socio-demographic criteria. All examiners completed all ratings, no missing data was observed. The study team’s initial ranking of students’ performances in the four videos was evident in the participants’ ratings as well. ‘Marit’ (M = 1.6, SD = 0.5) and ‘Anne’ (M = 1.7, SD = 0.5) received, on average, the best grades while both ‘Lars’ (M = 3.2, SD = 1.1) and ‘Kari’ (M = 4.1, SD = 1.4) were rated lower (cf. Figure 4). The average examiners-rated confidence in these grades were M = 75% (SD = 15%) and M = 79% (SD = 21%) for ‘Marit’ and ‘Anne’, respectively. The according ratings for ‘Lars’ were M = 71% (SD = 14%) and M = 65% (SD = 21%) for ‘Kari’. Across all performances, the examiners without experiences in the given exam rated their confidence with M = 63% (SD = 20%) while the examiners who had conducted this exam before rated theirs as M = 82% (SD = 11%). At the same time, the average grade across all performances for both those with and without was M = 2.7 (SD = 1.42).

### Inter-rater agreement using Fleiss Kappa

Overall agreement for both the pass/borderline/fail decisions and the grades on the A-F scale across the four candidates were Kappa_/Pass/Borderline/Fail_ = 0.07 and Kappa_A–F_ = 0.14. Kappa coefficients for experienced examiners (N = 5 with prior experience) were of similar magnitude with Kappa_/Pass/Borderline/Fail_ = 0.09 and Kappa_A–F_ = 0.15. Across conditions, Kappa coefficients generally indicated low to slight agreement.

### Within-examiner consistency in use of rating scales

For each participant, we calculated a rank-correlation (Spearman’s Rho) between the ratings-scales on demonstrated basic knowledge, applied knowledge and reasoning and the overall A–F grade given in the scoring rubric. On average, the intra-rater correlation among these elements of the scoring rubric was r = 0.79 (range: r = 0.50 to r = 1.00).

### Statistical analysis of differences in inconsistencies

**Fig. 5:**
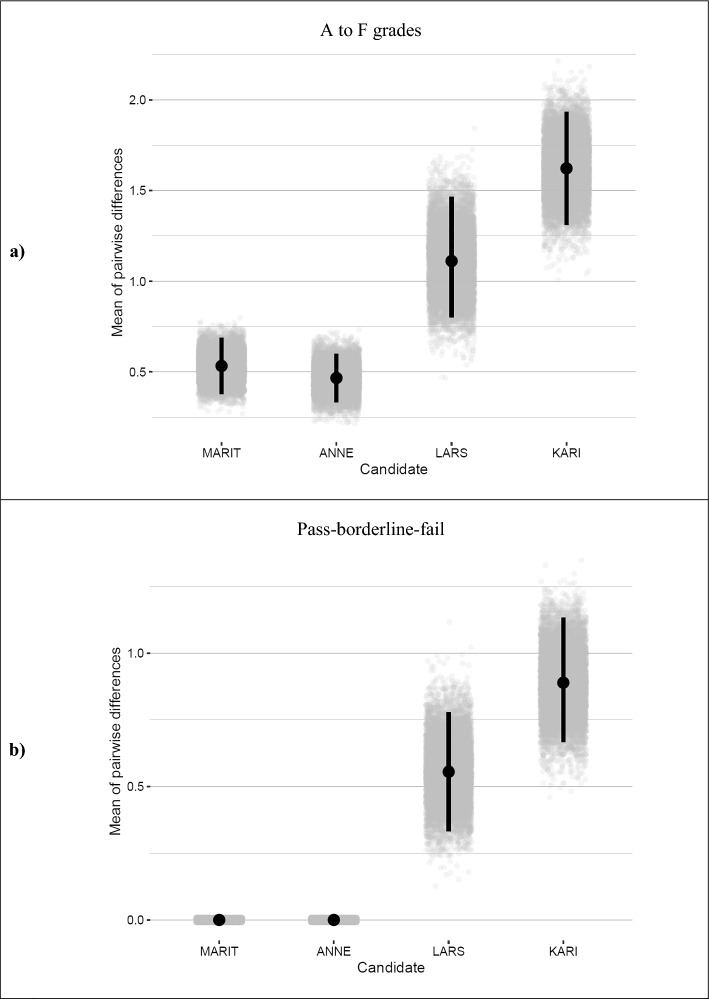
Statistical test of pairwise differences in **a** grades and **b** pass-borderline-fail decisions using non-parametric bootstrapping. Grey dots represent the average per one repretition in the bootstrap. Black points represent the average across 10.000 repetitions. Errorbars depict 95% bootstrap confidence limits. (Color figure online)

Results from our bootstrapping analysis indicate that the pairwise differences in ratings varied significantly across performance-levels (see Fig. 4a and b). The 95% bootstrapping interval for ‘Kari’ and ‘Lars’ (low-performing students) indicated significantly larger disagreement than the performances of ‘Anne’ and ‘Marit’ (high-performing students). This was true for both the A-F grades (Fig. 5a) and the pass-borderline-fail-classifications (Fig. 5b).

In detail, the analyses highlight that, using A-F grades, differences were on average half a grade for the two best performing students ‘Marit’ and ‘Anne’, meaning that these two candidates had a one-grade range in their evaluations. These differences were significantly lower than those for ‘Lars’ and ‘Kari’ who varied by 1.1, and 1.6 grades, respectively. Hence, these candidates had, on average, a range of discrepancies spanning two to three grades. This pattern was evident for the pass/borderline/fail classification, too. Both ‘Marit’ and ‘Anne’ were consistently classified as clear passes. Classifications for both ‘Lars’ and ‘Kari’ were significantly less consistent. Judgments on both performances showed differences in classifications larger than 0.5, which indicates that these two candidates, on average, are placed between two or more of the three categories.

### Collective decisions and simple heuristics

Using the combined decisions of all ten examiners as a gold standard resulted in pass-decisions for the four candidates when the *majority-rule* was employed. The results were identical when the *majority-rule* was used for either the most experienced half of the examiners or the five most confident ones.

For the examiner-pairs, results indicate that, in all conditions, the heuristics ratings of dyads exceeded the performance of the benchmark approach where verdicts were determined randomly. Furthermore, we find that decisions for the two best performing students are generally “pass”. Final verdicts for the less well performing students varied among the five heuristics employed. For ‘Lars’, the decision rule that most consistently reproduced the gold standard was the decision tree (> 90% pass). For ‘Kari’, results varied between 64.6 and 73.5% passes in the heuristic favouring the more experienced assessors and the confidence focused heuristic, respectively. All details from these analyses are given in Table 2.

**Table 2:** Percentages in which examiner-pairs replicate the full group verdict (i.e., “PASS”)

	MARIT	ANNE	LARS	KARI
0: Randomly pick decision from examiner-pair	100%	100%	80.1%	60.2%
1: Most confident → random	100%	100%	87.6%	73.5%
2: Most confident → most experienced → random	100%	100%	88.3%	73.5%
3: Most experienced → random	100%	100%	87.6%	64.6%
4: Most experienced → most confident → or random	100%	100%	87.3%	68.5%
5: Decision tree (Fig. 3)	100%	100%	91.1%	71.2%

## Discussion

Inconsistencies in judgments within rater-based assessments continue to be a matter of concern. In this study, our aim was to develop a better understanding of how a lack of agreement between judgements in examiner pairs can be pragmatically resolved. We furthermore took in consideration that ambiguity in judgments itself might be related to the level of candidate performance. In our study, we found that inconsistencies among examiners are largely a concern for candidates performing around the borderline level. In contrast to these levels, high-level performances were rated consistently. These results are in line with previous, typically more descriptive, findings (Eva, 2018; Holmboe et al., 2003; Larson & Billeter, 2017; Tweed & Ingham, 2010).

Furthermore, we focussed on probing an approach for how to remedy the observed discrepancies in rater judgment. Building on research in human decision making, we first collect judgments independently and then resolved possible ambiguity using simple decision rules. To this aim, we explored whether simple rules known from research on heuristic decision making would, when applied to judgments from pairs of examiners, help to improve decision making. All decision rules did approximate the verdict of the group of ten examiners, but we also found that the heuristics employed varied in the degree to which they did so. In conclusion, our results suggest that, in our scenario, heuristics seem to differ in their attributes, a property which needs more attention in future research.

To the best of our knowledge, this is the first study that applies this theoretical framework to data from assessments in health professions education. Thus, on a practical level, the implications should be considered with care. One clear implication, however, is that developing simple decision rules might be one further way to improve assessment, especially in contexts where other approaches such as sophisticated psychometric analyses or extensive systems of programmatic assessment might not be feasible. In addition, we here describe one specific context of application, a structured oral exam, which is different from many studies in related research in rater cognition (Gingerich et al., 2017; Yeates et al., 2013). Nevertheless, we argue that research on the application of decision rules could also be beneficial in other educational context, such as in programmatic assessment and competency based medical education.

From a theoretical perspective, we want to stress that the approaches used here have been well-researched in both medical and non-medical contexts (Gigerenzer & Goldstein, 1996; Kämmer et al., 2017; Kurvers et al., 2016; Litvinova et al., 2022). They help to increase diagnostic accuracy in a clinical context and thus could also be a valuable option to help to improve accuracy of decisions in assessment contexts. Here, we argue that our findings illustrate that such a perspective could be a valuable endeavour. Conceptually, we agree with the arguments put forward by Hautz et al. (2020) who elaborated on collaborative diagnostic decision-making in the clinical context: Given that many professional environments—surely education—are collaborative by nature, the important question is *when* and *why* various ways of using collaboration, aggregation, or independent reasoning in decision making are most appropriate. While Hautz and colleagues formulated this for research in diagnostic decision making, this challenge has not been fully embraced in research in rater-based assessment either.

An important limitation of our study is the lack of a clear failing candidate performance, and, more generally, that the number and variation of performances was limited. To provide a full evaluation of the heuristics used—in terms of both the sensitivity and specificity—we would have needed recordings of clear-fail performances. Put differently, our specific selection of performances is too narrow to evaluate the heuristics’ utility to improve the classification into either competent or incompetent. Still, our study also serves an illustrative purpose as we understand assessment as an act of human decision making. Given this perspective, we suggest that research into ecological rationality and heuristics offers the potential to improve such decision making in assessment contexts.

Another limitation is the sample size of the number of examiners involved. For instance, other studies draw on considerably larger pools of eligible experts or participants (Gingerich et al., 2017; Kämmer et al., 2017; Kurvers et al., 2016; Yeates et al., 2012). In our study, we assume that the participants involved are an adequate sample of the possible examiners in our educational setting. Going beyond this specific sample would probably have introduced additional sources of variation since those participants would not have been familiar with the actual curricular content. At the same time, similarly intensive studies employed between nine and 14 experts or examiners (Gingerich et al., 2017; Ginsburg et al., 2017; Mazor et al., 2007; Yeates et al., 2020). Indeed, exam boards, competence committees, or standard-setting committees are often of comparable group size as well.

The ecological validity of the approach presented here might be limited as examiner pairs in real context typically could interact directly after seeing a candidate perform. As discussed earlier, it is still an open and under-researched question under which circumstances such interaction might improve decision making. Hence, we should engage in research on if, when, and how interaction and aggregation of information improves decisions. Another limitation concerns our “benchmark” condition to which we compared outcomes of the decision rules applied here. Future research might consider letting actual rater-pairs interact and select a decision and use this as a comparison for heuristics. Similarly, we opted for a random draw of a pass-fail decision in the case that disagreement cannot be resolved. Indeed, another option would have been to generally pass or fail candidates if there is disagreement between raters. While indeed important, a thorough discussion of the according rationale and justification is beyond the scope of this paper.

Despite these limitations, we argue that our study contributes to the research on rater-based assessment in multiple, critical ways. First, we provide empirical evidence of the relationship between examiner discrepancies and performance-levels in a systematic and statistically tested manner and corroborate earlier, descriptive, findings. This has implications for research on rater-cognition and expert-judgment where clarity and ambiguity of the task at hand has rarely been taken explicitly into account. Second, we highlight that the framework of ecological rationality can not only contribute to understanding examiner discrepancies, but also offers approaches for how to resolve the ambiguity of such judgments. Finally, we point to directions not only for further research but also for development of assessment practices that can be implemented in medical schools.

Clearly, there are aspects of our study that could be extended further—for instance investigating the effects of increasing the number of examiners from two to three and further. Furthermore, applying algorithms to determining grades instead of pass/fail decisions might be an interesting endeavour. More generally, it might be a promising area of research and development to focus on the use of decision-trees for assessments, rather than checklists or rating scales. Clearly, these tools, to be defensible, need to be based on expert committees and/or empirical evidence. In this respect, we hope to stimulate research and development which helps to further improve the quality of assessment in the health professions.

In conclusion, we do not propose that we provide generic answers to a common issue in rater-based assessment. Rather, we point at a rich body of literature in both medical and non-medical decision making which has, in our opinion—and given the findings in this study—great potential for improving decision making in assessment contexts, too.
